# A Novel 2D-to-3D Video Conversion Method Using Time-Coherent Depth Maps

**DOI:** 10.3390/s150715246

**Published:** 2015-06-29

**Authors:** Shouyi Yin, Hao Dong, Guangli Jiang, Leibo Liu, Shaojun Wei

**Affiliations:** Institute of Microelectronics, Tsinghua University, Beijing 100084, China; E-Mails: h-dong12@mails.tsinghua.edu.cn (H.D.); jgl12@mails.tsinghua.edu.cn (G.J.); liulb@tsinghua.edu.cn (L.L.); wsj@mail.tsinghua.edu.cn (S.W.)

**Keywords:** TV and home gaming equipment, stereoscopic video, 2D-to-3D video conversion, time-coherent depth maps

## Abstract

In this paper, we propose a novel 2D-to-3D video conversion method for 3D entertainment applications. 3D entertainment is getting more and more popular and can be found in many contexts, such as TV and home gaming equipment. 3D image sensors are a new method to produce stereoscopic video content conveniently and at a low cost, and can thus meet the urgent demand for 3D videos in the 3D entertaiment market. Generally, 2D image sensor and 2D-to-3D conversion chip can compose a 3D image sensor. Our study presents a novel 2D-to-3D video conversion algorithm which can be adopted in a 3D image sensor. In our algorithm, a depth map is generated by combining global depth gradient and local depth refinement for each frame of 2D video input. Global depth gradient is computed according to image type while local depth refinement is related to color information. As input 2D video content consists of a number of video shots, the proposed algorithm reuses the global depth gradient of frames within the same video shot to generate time-coherent depth maps. The experimental results prove that this novel method can adapt to different image types, reduce computational complexity and improve the temporal smoothness of generated 3D video.

## 1. Introduction

3D display is now one of the most attractive emerging display technologies and is regarded as the most promising display technology in the future. 3D display based on parallax technology is entering the consumer markets, especially the entertainment market. This technology has been used in various entertainment applications, such as movies, computer games, and animation. 3D entertainment is now becoming more and more popular and it will be the future trend in the field of entertainment. However, one of the challenges that has been faced in the field of 3D entertainment is the lack of 3D video content.

The approach to obtain 3D video content requires professional stereoscopic camera containing two-view image sensors. However, professional equipment is cumbersome, expensive and difficult to operate [1]. These limitations prevent wide use of this method. Consequently, there is not sufficient 3D video content in the market to meet the consumers’ demands for 3D entertaiment.

**Figure 1:**
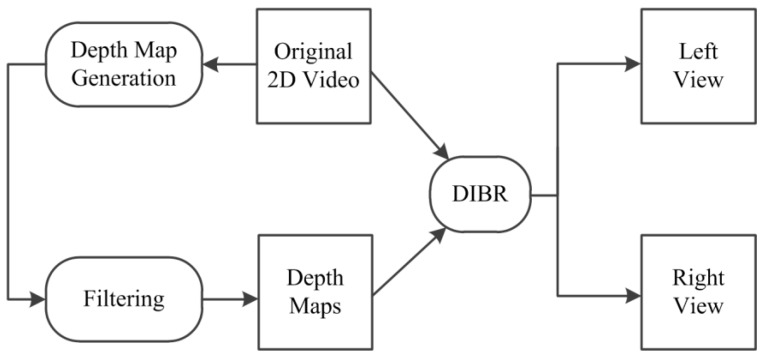
The framework of 2D-to-3D video conversion.

As 2D image sensors are very common around us, making full use of an ordinary single 2D image sensor to generate 3D video content has broad prospects. It is challenging but feasible: a new method using a 2D image sensor plus a 2D-to-3D conversion function can be adopted to obtain 3D video contents. An ordinary 2D image sensor can be packeged together with a 2D-to-3D chip to create a 3D image sensor. Thus the 3D video contents can be easily generated through 3D image sensors and the cost of acquisition of 3D video content can be reduced by a large margin, which will greatly promote the development of 3D entertainment markets. In this paper, we propose a new 2D-to-3D conversion algorithm, which can be adopted in a 3D image sensor. The framework of 2D-to-3D video conversion is shown in Figure 1.

In the process of 2D-to-3D video conversion, depth map generation is an essential procedure and the latter procedure is relatively fixed. 2D video has a lack of depth information compared with 3D video. Depth information can be depicted by a depth map, which is a gray-scale image whose value for the farthest pixel is 0 and the nearest pixel is 255. A depth map can be obtained from single monocular view through various depth cues including texture gradient, geometric perspective, focus/defocus, interposition, motion parallax, relative height or size and so on [1]. A number of methods have been proposed. Jung *et al.* [2] assigned depth map by relative height based on a line tracing method and depth refinement filter. Ideses *et al.* [3] generated a depth map from motion information provided in H.264 bit streams. Yamada *et al.* [4] use three depth scene models and color theory to produce a depth map. However, these methods are not adaptable to different image types and may produce temporal flicker as they ignore the temporal coherence of depth maps between frames in the original 2D video.

In this paper, we present a novel algorithm to overcome the above shortcomings. The algorithm first detects whether the input frame belongs to a new video shot or not. If the frame belongs to a new video shot, the algorithm judges its image type and assigns a global depth gradient according to its image type. Three categories of image types are defined in our algorithm: landscape type, linear perspective type and normal type. An approach to generate global depth gradient for each image type is devised as well. If the frame does not belong to a new video shot, the global depth gradient of previous frame is reused. After the global depth gradient is obtained, it is refined by local depth information to generate the depth map. Cross bilateral filter and DIBR are then applied to produce the final depth map and 3D video. The innovations of our work can be summarized in the two following points:

- The algorithm can determine the image type of the input frame and obtain the global depth gradient accordingly. Thus it has a good adaptability to different image types.
- The algorithm detects the video shot and reuses the global depth gradient of frames within the same video shot to produce time-coherent depth maps, which reduces computational complexity and promotes the temporal smoothness of generated 3D video.

The rest of this paper is organized as follows: Section 2 describes the proposed algorithm in detail. Section 3 gives the experimental results. Finally, concluding remarks are made in Section 4.

## 2. Proposed Algorithm

In the 2D-to-3D system, the procedure of depth map generation is essential. This algorithm thus mainly devises the depth map generation method. As for the procedure of filtering and DIBR, we adopt general methods. 

**Figure 2:**
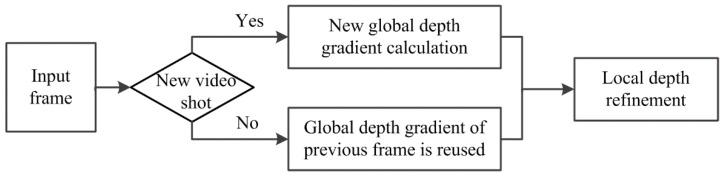
Flowchart of the depth map generation method.

An input 2D video stream consists of a number of video shots and each video shot includes a sequence of frames taken using a single camera. As the camera moves slightly within a video shot, the global depth gradient is changed slightly and it can be shared by frames in the same video shot to reduce the computational complexity and promote the temporal smoothness of the depth maps with little extra inaccuracy, which is proved by our experimental results in Section 3. Whether the input frame of video stream belongs to a new video shot is first detected. If the input frame is a new video shot frame, image type of this frame is judged and the global depth gradient is assigned accordingly. If the input frame is not a new video shot frame, the global depth gradient of the previous frame is directly adopted. That is, the global depth gradient is calculated from the first frame of a video shot and reused in other frames within the same video shot. The local depth information of the input frame is then utilized to generate the depth map. Figure 2 illustrates the flowchart of the depth map generation method.

### 2.1. New Video Shot Frame Detection

An input video stream is composed of a series of video shots. Video shot changes come in different types such as hard and gradual. A cut is a hard shot change which occurs in a single frame. Gradual type changes come in many forms including dissolve, wipe, fade in and fade out, and are processed not in a single frame but through several frames. The features of two frames spanning a cut have more differences while the adjacent frames of gradual type have fewer differences.

There are many methods of detecting a new video shot frame based on computing frame differences, such as pixel differences, statistical differences, edge differences and so on [5]. Color histogram difference is adopted in our method for the following reasons: first, a color histogram is sensitive to video shot boundary; second, it is not sensitive to object motion because the spatial changes between two frames do not affect its color distribution. HSI color space is used in our method as it is defined according to human color perception. As the human visual system cannot distinguish all the possible colors, there is no need to use all the colors to compute the histogram. A number of 1024 possible colors are used in the color histogram computation, including 16 levels for hue component, 8 levels for saturation component and 8 levels for intensity component [6]. The formula of color histogram is defined as follows:

(1) $$h\left(i\right)=\frac{n_{i}}{N},\;(i=1,2,\;...,\;1024)$$

**Figure 3:**
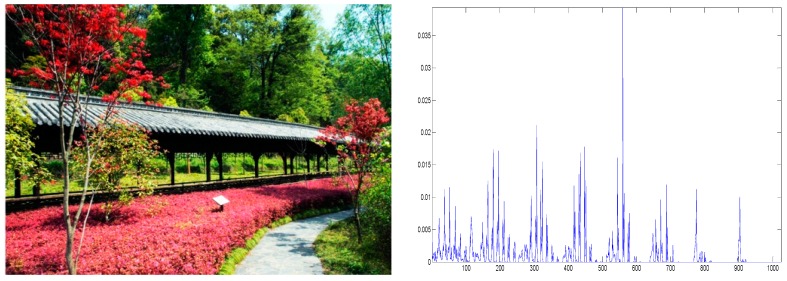
An image and its color histogram.

N is the total number of pixels of an image, $n_{i}$ is the number of pixels with color i. An image and its color histogram are shown in Figure 3.

As is shown in Equation (2), the color histogram difference is calculated between the input frame j and the previous frame j−1.

(2) $$HistDiff_{j}=\sum_{i=1}^{1024}\left|h_{j}\left(i\right)-h_{j-1}\left(i\right)\right|$$

**Figure 4:**
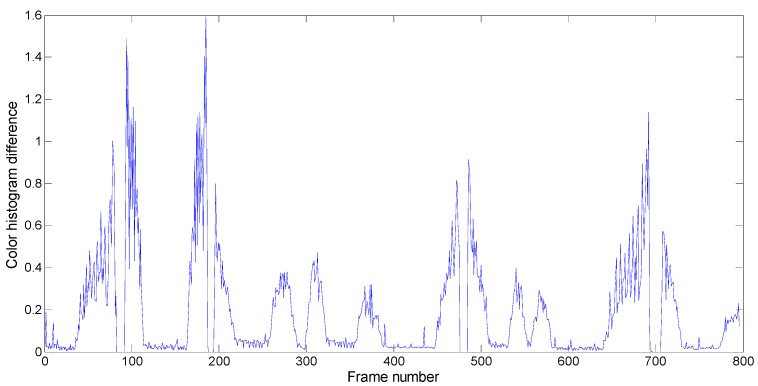
Frame-to-frame color histogram differences map of the tested video sequence.

$h_{j}\left(i\right)$ denotes the color histogram value of the color i in frame j. $HistDiff_{j}$ is the color histogram difference between frames j and j−1. The frame-to-frame color histogram differences map of the video sequence “Healing Soul” is shown in Figure 4.

The frame of a hard shot can be easily distinguished by the color histogram difference because the change of color histogram between two adjacent frames spanning a cut is very obvious. The method of adaptive twin thresholds for video shot frame detection has already been proposed and proved effective [7,8,9], so we adopted the method of adaptive twin thresholds to detect new video shot frames. The adaptive twin thresholds include a higher threshold $T_{H}$ and a lower threshold $T_{L}$. The high threshold $T_{H}$ can be determined to distinguish a cut. In order to detect a gradual shot frame, the low threshold $T_{L}$ is set to select the candidate start frame of gradual type shot. If the color histogram difference between frame j and j−1 is lower than $T_{H}$ but higher than $T_{L}$, frame j is set as candidate start frame $F_{C}$. The color histogram differences between $F_{C}$ and subsequent frames are calculated. If the value of the difference between $F_{C}$ and a frame $F_{I}$ is higher than $T_{H}$, frame $F_{I}$ can be identified as a gradual shot frame. If a new gradual shot frame is detected, the candidate start frame $F_{C}$ can be cancelled and the above process is continued to detect the next new shot frame [7].

We make use of a sliding window to determine the adaptive thresholds. The sliding window is from the previous video shot frame to the frame before the input frame, and the length of the sliding window is L. L is not a fixed value; it depends on the number of frames from the previous video shot frame and the number of the input frame. Average frame-to-frame color histogram difference ${\text{μ}}_{Histdiff}$ of the sliding window is calculated by the following equation:

(3) $${\text{μ}}_{Histdiff}=\frac{1}{L}\sum_{i=N}^{M}HistDiff_{i}=\frac{1}{M-N+1}\sum_{i=N}^{M}HistDiff_{i}$$

N is the first frame number of the sliding window, that is, N is the previous video shot frame number. M is the number of the frame before the input frame. $T_{H}=5\mu_{Histdiff}$ and $T_{L}=3\mu_{Histdiff}$ are adopted in the method of adaptive twin thresholds [8,9].

### 2.2. Global Depth Gradient Generation

If an input frame belongs to a new video shot, the global depth gradient is computed through the frame and reused in other frames within the same video shot. Global depth gradient is computed according to the image type. Three categories of image types are defined in our algorithm: landscape type, linear perspective type and normal type. Landscape type refers to the images of outdoor scenery largely comprising land areas, water bodies and sky. Image of linear perspective type contain mainly vanishing lines which converge into a vanishing point. Other images belong to the normal type, such as indoor images, close-up images, personal images and so on.

For a new shot frame, the algorithm first judges if it belongs to the landscape type. The landscape type is generally portrayed such that the upper part of the image is the sky and the lower part is the ground or water body such as a lake, river or sea. HSI color space was already transformed in the previous process of new shot frame detection and it is also used to detect the landscape type. A simple but effective judgement is adopted, as is shown in Equation (3). If the intensity value of a pixel is between 80 and 255 and its hue value is between 100 and 180, the pixel may belong to the sky or water body. If the saturation value is between 80 and 255 and its hue value is between 20 and 100, the pixel may belong to the ground [10]. The judgement equation is as follows:

(4) $$LS(x,y)=\left\{ \begin{array}{l} 1,\;if((80<I(x,y)<255)\&\&(100<H(x,y)<180))\\ \;||\;((80<S(x,y)<255)\&\&(20<H(x,y)<100))\\ 0,\;otherwise \end{array}\right.$$

where H(x,y), S(x,y) and I(x,y) are the hue, saturation and intensity of pixel (x,y). LS(x,y)means that the pixel (x,y) belongs to the physical elements of landscape type including the sky, water body or the ground. As is shown in Equation (5), the total amount of pixels belonging to the physical elements of landscape type is computed in an image. X and Y denote the height and width of the image.

(5) $$Amount=\sum_{x=1}^{X}\sum_{y=1}^{Y}\left(LS(x,y)\right)$$

As is shown in Equations (6) and (7), the proportion of the pixels belonging to the physical elements of the landscape in an image is computed and compared to the threshold *T_Ls_*. The threshold *T_Ls_* is defined heuristically by experimental analysis as 0.5.

(6) $$prop=\frac{Amount}{X\times Y}$$

(7) $$Image=\;\left\{ \begin{array}{l} landscape\;type,\;if\;(Prop>T_{LS})\\ non-landscape\;type,\;if\;(Prop\leq T_{LS}) \end{array}\right.$$

**Figure 5:**
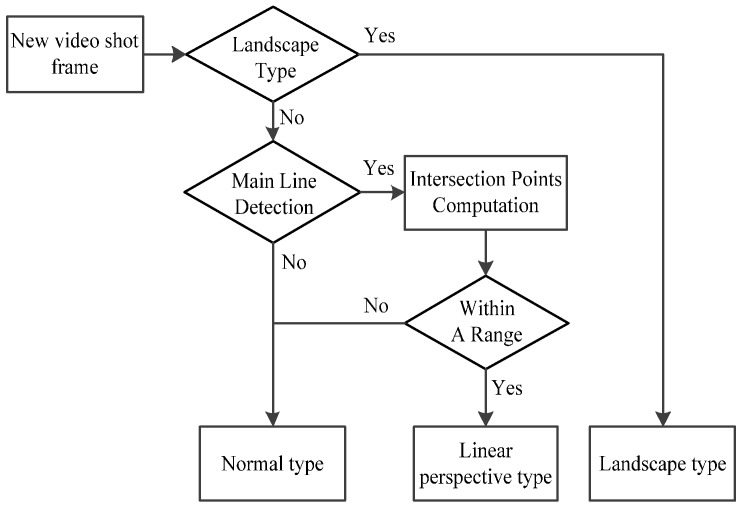
Flowchart of image type judgement.

If the frame does not belong to the landscape type, the algorithm then judges if the image falls into the linear perspective type by the following procedure: a Hough transform is used to detect straight lines and a threshold is set to obtain main lines from the straight lines. If there are main lines, the intersection points of every two lines are computed. The algorithm also checks if these intersection points are within a predefined range. If so, the input frame can be classified as linear perspective type. The intersection point nearest to the central point of these points can be regarded as the vanishing point and the main lines converging to the vanishing point are regarded as vanishing lines. If the main lines are not detected or the intersection points are decentralized, the frame is classified as normal type. Figure 5 shows the above process.

**Figure 6:**
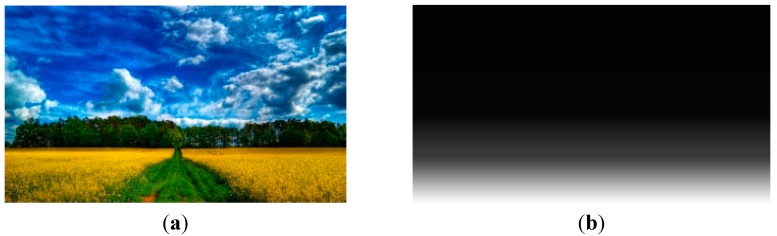
(**a**) Image of landscape type; (**b**) global depth gradient of the image.

After the image type of input frame is determined, global depth gradient is generated accordingly. For the landscape type, the upper part of the image is the sky and the lower part is the ground or water. Cumulative horizontal edge histogram [11] is used to assign global depth gradient for the following reason: As cumulative horizontal edge histogram represents the horizontal edge complexity and the sky is often smoother than the ground or water, there is a distinct depth change between the sky and the ground or water in an image of landscape type. Besides, as the global depth gradient is roughly far-to-near from top to bottom, it can be assigned 0 to 255 from top to bottom by a normalizing cumulative horizontal edge histogram. Figure 6 shows an image of landscape type and its global depth gradient.

**Figure 7:**
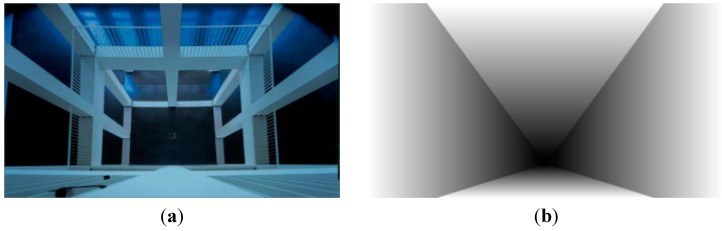
(**a**) Image of linear perspective type; (**b**) global depth gradient of the image.

For the linear perspective type, the vanishing point is considered to be the farthest point in the image. The vanishing lines divide the image into horizontal planes and vertical planes. The depth gradients in different planes are assigned separately. In horizontal planes, the depth gradient value is constant along the rows. In vertical planes, the depth gradient is constant along the columns [12]. Figure 7 shows an image of linear perspective type and its global depth gradient.

For the normal type, numerous edges representing abundant details attract viewer’s attentions. Consequently, a closer depth is assigned to the regions containing more edges. The main steps are as follows:

(1) Divide the image into N blocks: $B_{1},B_{2},\;...,\;B_{N}$. Calculate the number of edges for each block: $E_{1},E_{2},\;...,\;E_{N}$. Parameter N is set to 16 in the proposed algorithm so we divide the image of normal type into 4 lines and 4 columns.
(2) A threshold $E_{av}$ is set to select the main blocks. $E_{av}$ is the average edge amount in a block of the total edges, which is shown in Equation (8).
  (8) $$E_{av}=\frac{\sum_{i\text{=}1}^{N}E_{i}}{N},\;\left(i=1,2,\;...,N\right)$$
  If the number of edges $E_{i}$ (i=1,2, ...,N) of the block $B_{i}$ (i=1,2, ...,N) is larger than $E_{av}$, the block $B_{i}$ can be regarded as main block. If the edge amount $E_{i}$ (i=1,2, ...,N) of the block $B_{i}$ (i=1,2, ...,N) is smaller than $E_{av}$, the block $B_{i}$ can not be regarded as main block. The number of main blocks is T and the number of edges in each main block is $E_{T1},E_{T2},\;...,\;E_{TT}$.
(3) Make use of the main blocks individually, assign T branching global depth gradients $G_{T1},G_{T2},\;...,\;G_{TT}$. For every $G_{Ti}(i=1,\;2,\;...,\;T)$, the centre point of the selected main block is regarded as the nearest point and $G_{Ti}$ is assigned according to the Euclidian distance between each point of the image and the centre point.
(4) Then fuse them together by the weight of $W_{T1},W_{T2},\;...,\;W_{TT}.$

**Figure 8:**
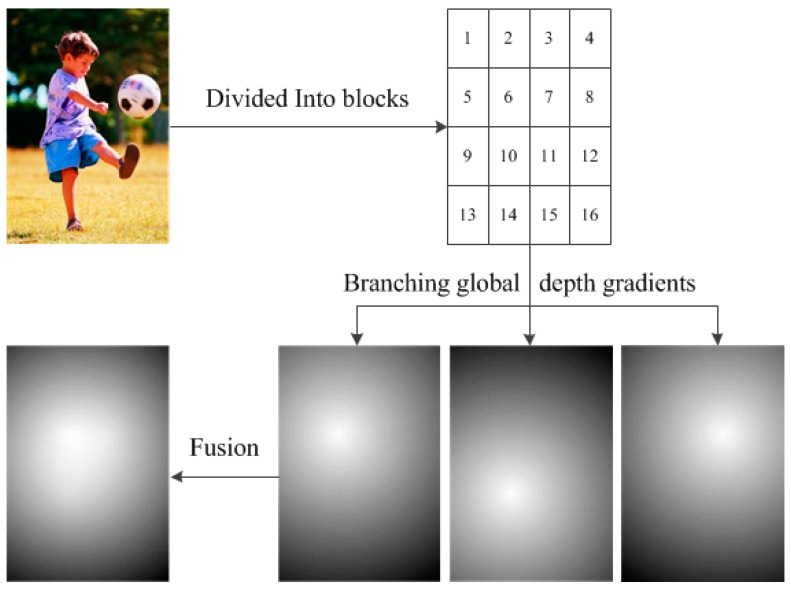
Global depth gradient generation of normal type image.

As is shown in Equations (9) and (10), the weights and the fused global depth gradient are calculated. Figure 8 shows the procedure.

(9) $$W_{Ti}=\;\frac{E_{Ti}}{\sum_{i=1}^{T}E_{Ti}},\;(i=1,\;2,\;...,\;T)$$

(10) $$D_{global}\text{=}\sum_{i=1}^{T}W_{Ti}\times G_{Ti}$$

### 2.3. Local Depth Refinement

After the global depth gradient of input frame is obtained, it is then refined by local depth information to generate a depth map. Warm/cool color theory suggests that the warm color gives viewer a nearer feeling while cold color is farther in visual perception [11] so the color information is adopted to refine the global depth gradient. The Y and Cr components of YCbCr-color space are used in the following calculation. As is shown in Equation (11), the depth map can be obtained by fusing global depth gradient and color information.

(11) $$D_{f}=\alpha \times D_{global}+\beta \times D_{Cr}+(1-\alpha -\beta )\times D_{Y}$$

**Figure 9:**
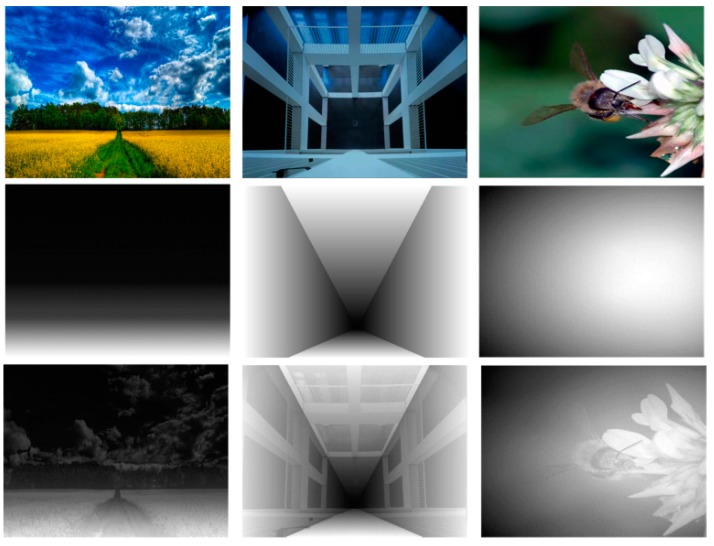
Original image, global depth gradient and depth map of landscape type (first column), linear perspective type (second column) and normal type (third column).

Cr and Y components of the YCbCr-color space are normalized as $D_{Cr}$ and $D_{Y}$. $D_{global}$ is the global depth gradient and $D_{f}$ is the obtained depth map after fusion. α is the weight for *D_global,_*, β is the weight for $D_{Cr}$ and (1−α−β) is the weight for $D_{Y}$. α and β are defined heuristically by experimental analysis as 0.6 and 0.2 respectively. Figure 9 shows the original images, global depth gradients and depth maps of different image types.

### 2.4. Depth Map Smoothing & Depth-Image-Based-Rendering (DIBR)

The procedure of filtering and DIBR are relatively fixed, so we adopt general methods to implement them. Depth map smoothing can reduce the number of holes generated during the virtual view synthesis in the DIBR procedure and help to improve the image quality of virtual views [13]. Gaussian filter is used in our algorithm, which is shown in the following equation:

(12) $$g(x,\sigma )=\frac{1}{\sqrt{2\pi }\sigma }\exp\left\{-\frac{x^{2}}{\sigma^{2}}\right\},\;-\frac{w}{2}\leq x\leq \frac{w}{2}$$

w is the filter’s window size and σ is the standard deviation. The standard deviation determines the depth smoothing strength.

(13) $$D(x,y)=\frac{\sum_{v=-\frac{w}{2}}^{\frac{w}{2}}\left\{\sum_{\mu =-\frac{w}{2}}^{\frac{w}{2}}\left(D_{f}(x-\mu ,y-v)g(\mu ,\sigma_{\mu }))g(v,\sigma_{v}\right)\right\}}{\sum_{v=-\frac{w}{2}}^{\frac{w}{2}}\left\{\sum_{\mu =-\frac{w}{2}}^{\frac{w}{2}}g(\mu ,\sigma_{\mu })g(v,\sigma_{v})\right\}}$$

$D_{f}(x,y)$ is the depth value in depth map at the pixel (x,y). D(x,y) is the final depth value by using Gaussian filter.

**Figure 10:**
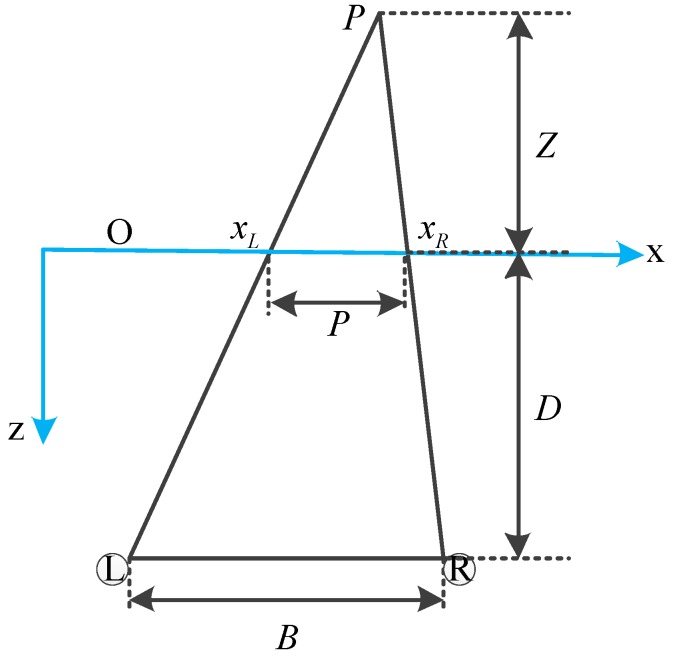
Model of a stereoscopic viewing system.

Depth-Image-Based-Rendering (DIBR) is then processed to generate stereoscopic video content. There are three steps of DIBR process: disparity computation, pixel-shifting and hole-filling [1]. Disparity can be calculated based on depth value. Figure 10 shows the model of a stereoscopic viewing system and the calculation procedure is shown in Equations (14) and (15).

(14) $$\frac{P}{B}=\frac{-Z}{D-Z}$$

(15) $$P=x_{R}-x_{L}=B\left(\frac{-Z}{D-Z}\right)$$

P denotes the horizontal disparity between left image and right image on the screen. B corresponds to the distance between the two eyes and D represents the viewing distance from the screen. Z is the perceived depth value of a pixel, which can be mapped from the final depth value.

Pixel-shifting renders a virtual image by projecting the pixels of the original viewpoint to another viewpoint according to calculated disparity. Due to the disocclusions appear in another viewpoint, the hole-filling process is to fill in the newly exposed areas to form a complete image in the virtual viewpoint. After the two-view video content is produced through DIBR procedure, we can make use of 3D display technologies to enjoy 3D video. 3D display technologies include anaglyph 3D display, polarization 3D display and active-shutter 3D display.

## 3. Experimental Results

In this section, we first give some experimental results of different important parameters used in the proposed algorithm, then we designed different experiments to evaluate the proposed algorithm. To evaluate the adaptability of the proposed algorithm to different image types, various video sequences such as “Timescapes” and “Country Road” were tested as they contain abundant scene types. To evaluate the benefit of the reuse of the global depth gradient in the proposed algorithm, calculation instead of reuse of global depth gradient in each frame within the same video shot is also conducted in our experiment. By comparing them in terms of similarity and processing time, we can evaluate the reasonableness and necessity of the reuse of the global depth gradient. To evaluate the advantages of the proposed algorithm, three algorithms were adopted as reference. Average processing time and subjective testing outcome are compared between them.

### 3.1. Important Parameters Determination

To determine the threshold *T_Ls_* for the image type of landscape, we tested 120 pictures from the Imagenet database. Sixty pictures belong to landscape type and sixty pictures do not belong to landscape type. Precision rate, recall rate and F score are often used to evaluate the effectiveness of method for classification [14]. Table 1 shows different values of thresholds and corresponding precision rates, recall rates and F scores. From Table 1 we can find that when 0.5 is selected as the value of threshold *T_Ls_*, the F score can reach the highest value among these values. Consequently, we choose 0.5 as the value of threshold *T_Ls_*.

**Table 1:** Different values of thresholds and corresponding precision rates, recall rates and F scores.

*T_Ls_*	0.3	0.4	0.5	0.6	0.7	0.8
Precision rate	72.2%	82.2%	89%	94.4%	92.5%	100%
Recall rate	100%	100%	95%	85%	61.7%	43%
F score	0.84	0.90	0.92	0.89	0.74	0.60

**Figure 11:**
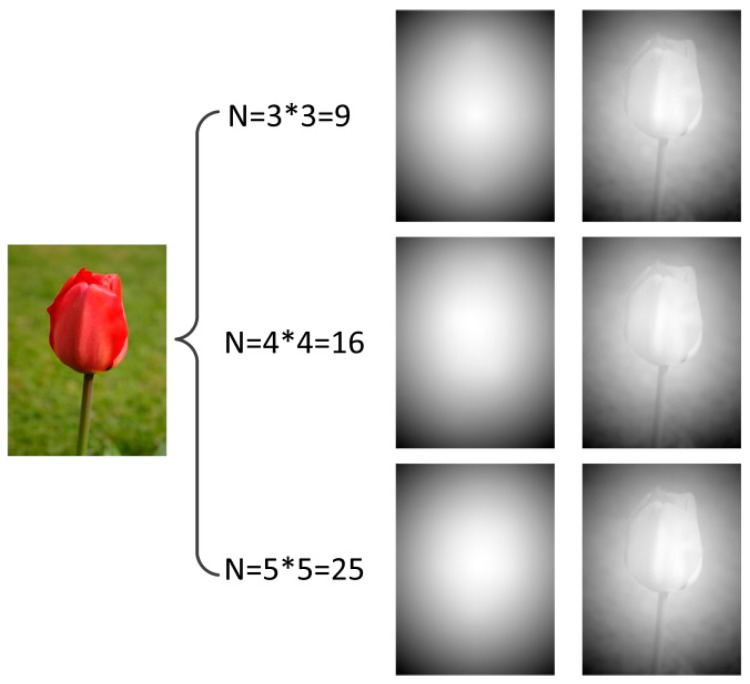
Comparison of global depth gradients and final depth maps of different N value.

For the normal type, the image is firstly divided into N blocks. The value of 4 × 4 is adopted as the parameter N in our experiment. Figure 11 shows the global depth gradients and the final depth maps when the parameter N is adopted as 3 × 3, 4 × 4 and 5 × 5.

From the comparision we can find that when N is adopted as 4 × 4, the effect of global depth gradient and final depth map is as good as N equal to 5 × 5 and the effects are both better than the N equal to 3 × 3. However, when N is adopted as 4 × 4, there are less branching global depth gradients and the computational complexity is less than N equal to 5 × 5. Consequently, the parameter N is set to 4 × 4 in the proposed algorithm.

**Figure 12:**
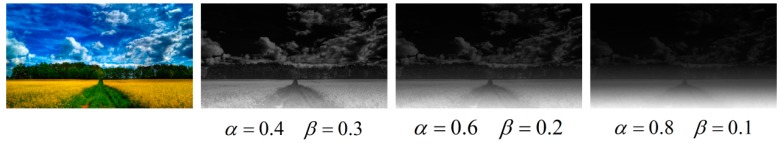
Comparison of depth maps of different α and β values.

For the fusion of global depth gradient and local depth information, we should determine the values of α and β. Because the global depth gradient is the major depth information in the proposed algorithm and the local depth information is adopted to refine the global depth gradient, α is larger than β in the proposed algorithm. Besides, the depth information from Cr and the depth information from Y have the same status, so β and (1−α−β) are equal in our experiment. Figure 12 shows an example of different depth maps when the weights of α and β are different values.

We can find that when α is 0.6 and β is 0.2, the final depth map has a better effect and is closer to depth perception by human eyes.

### 3.2. Adaptability to Different Image Types of Video Shots

**Figure 13:**
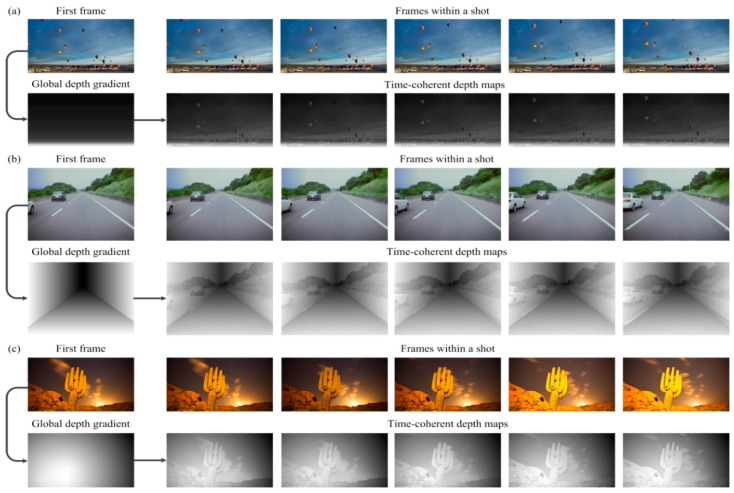
Input frames within a video shot, corresponding global depth gradient and depth maps. (**a**) Landscape type; (**b**) linear perspective type; (**c**) normal type.

Figure 13 shows the frames of video shots in different image types, corresponding global depth gradients and the time-coherent depth maps. As is illustrated, the global depth gradient is calculated by the first frame of the video shot and reused in other frames within the same video shot. For each frame, the global depth gradient is supplemented with local depth refinement to generate the depth map. The proposed algorithm can judge the image type of the input frame and then generate the global depth gradient accordingly, which improves adaptability to different image types. We find that the proposed algorithm can produce good depth maps for all of the three scene types.

### 3.3. Reuse of Global Depth Gradient within the Same Video Shot

**Figure 14:**
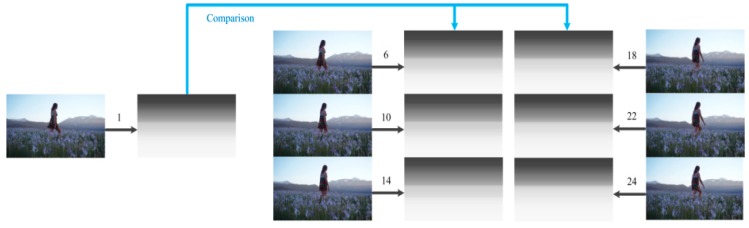
Comparison between the reused global depth gradient and the calculated global depth gradients in frames with a video shot.

The reuse of global depth gradient is another innovation point in the proposed algorithm. As the camera moves slightly within a video shot, the image type of these frames within a video shot does not change and the global depth gradient is changed only slightly during the generation process. As a result, there is no need to calculate the global depth gradient in every frame. Reuse of the global depth gradient within the same video shot brings the benefits of saving time and promoting temporal smoothness with little added inaccuracy. In the experiment, we calculated global depth gradient for every frame in a video shot and then compared them with the reused global depth gradient calculated in the first frame of this video shot, as is shown in Figure 14. The number in Figure 14 represents the frame number in the video shot.

As the range of value for depth map is from 0 to 255, a tiny change in value of a pixel such as 10 or 15 in a depth map can be acceptable. Although we cannot distinguish the difference with our eyes in the above figure, we computed the similarity between the calculated and reused global depth gradient of the frames within a video shot. The similarity value between two gray images is a percentage which divides the number of pixels whose value changes are under a tolerant value by the number of total pixels in an image. We set the tolerant value change as 10 and 15 respectively.

**Figure 15:**
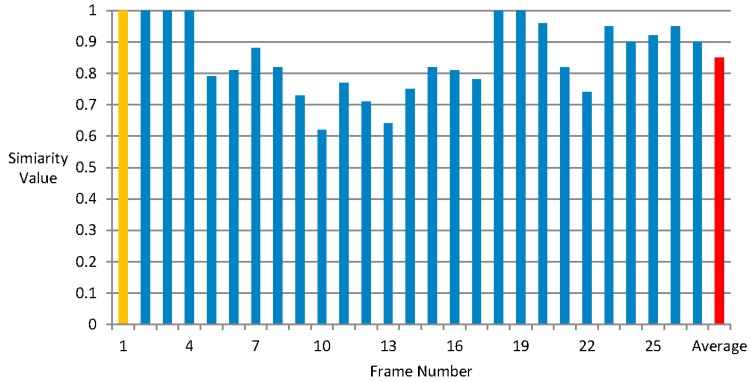
The similarity values of the global depth gradient calculated in the new video shot frame and the global depth gradients in frames with a video shot under tolerant value 10.

**Figure 16:**
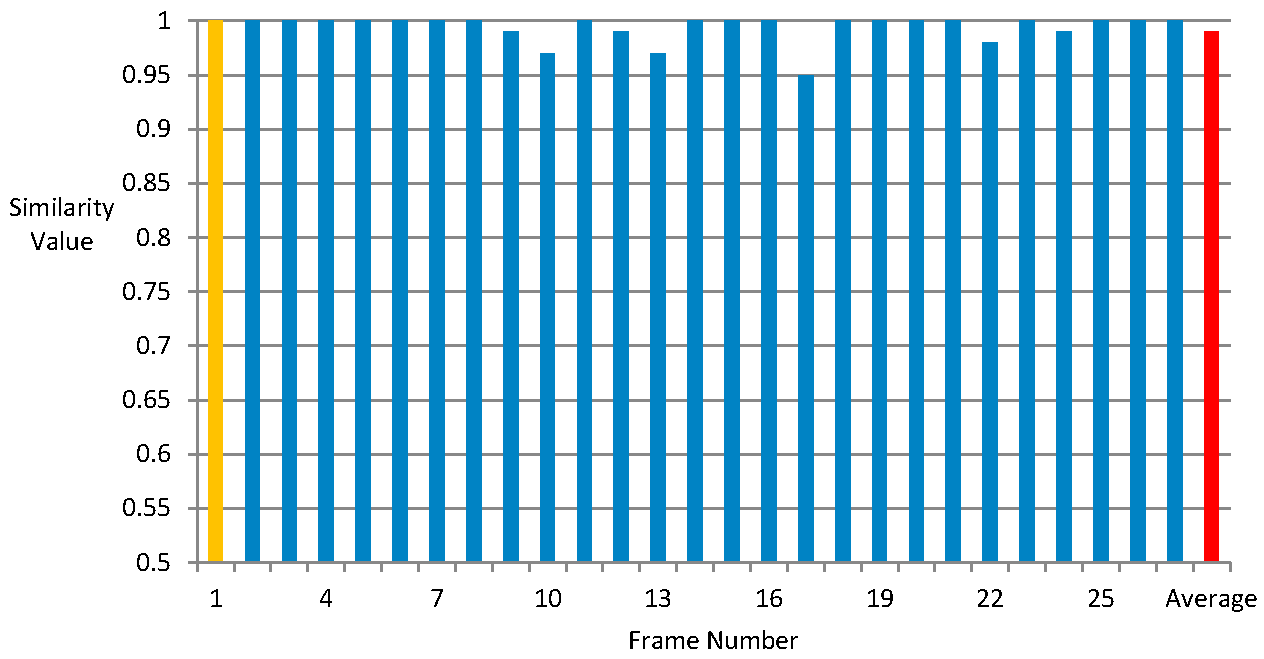
The similarity values of the global depth gradient calculated in the new video shot frame and the global depth gradients in frames with a video shot under tolerant value 15.

Figure 15 and Figure 16 show that the similarity values of the global depth gradient calculated in the new video shot frame and the global depth gradients in frames with a video shot under tolerant values 10 and 15 respectively. The horizontal axis is the frame number within the same video shot and the vertical axis is the similarity value.

From Figure 15 and Figure 16, we can find that the reuse of global depth gradient does not lead to much inaccuracy. The similarity is up to 85% and 99% when the tolerant value change is 10 and 15. The price of reusing the global depth gradient is tiny, so it is reasonable to reuse the global depth gradient. Besides, this innovation can bring much benefit of saving processing time.

To make a comparison of processing time, we processed every frame by the procedure of global depth gradient calculation, local depth refinement, filtering and DIBR without the reuse of the global depth gradient. Average processing time of above procedure is listed as procedure 1 in Table 2 and procedure of the proposed algorithm which reuses the global depth gradient is listed as procedure 2. The experimental platform is a PC with Intel quad-core i5 CPU and 4GB RAM. Spatial resolution of tested videos is 1280 × 720.

**Table 2:** Comparison of average processing time for each procedure.

Procedure 1	Average Processing Time (ms/frame)	Procedure 2	Average Processing Time (ms/frame)
Global depth gradient calculation	267	New video shot frame detection	25
		Global depth gradient generation	8
Local depth refinement	13	Local depth refinement	13
Filtering & DIBR	32	Filtering & DIBR	32

From Table 2 we can find that the calculation of global depth gradient takes up too much processing time. After we reuse the global depth gradient within the same video shot, average processing time of global depth gradient generation can be reduced greatly while the added procedure of new video shot frame detection does not take up too much time, and thus the overall processing time of 2D-to-3D system can be saved. Consequently, the innovation of reuse of global depth gradient is reasonable and necessary.

### 3.4. Comparison of Different Algorithms

**Figure 17:**
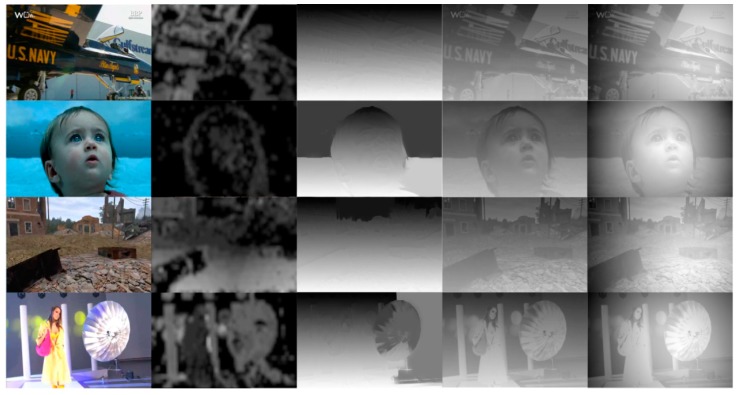
Original 2D images (first column), generated depth maps of [16] (second column), [17] (third column), [18] (fourth column) and the proposed algorithm (fifith column).

To evaluate the proposed method, we also compare the proposed algorithm with other algorithms. Four video sequences, “Air”, “Arctic”, “Fashion” and “Cod” from [15], are tested. The algorithms [16,17,18] are adopted as references. Figure 17 shows the original 2D images and generated depth maps of these algorithms. We can find that the proposed algorithm has advantages in terms of accuracy of the generated depth maps. The algorithm as in [16] relies on the motion vector. If an object in the image does not have relative motion, the depth cannot be extracted correctly. The edge-based algorithm in [17] will be not accurate when the foreground object is large. The algorithm as [18] cannot adapt to different scene types compared to the proposed algorithm. Besides, the latter two algorithms use only a single image to generate depth maps and ignore the temporal coherence of depth maps between frames in the original 2D video, thus they may produce temporal flickering and redundant computation in the process.

**Figure 18:**
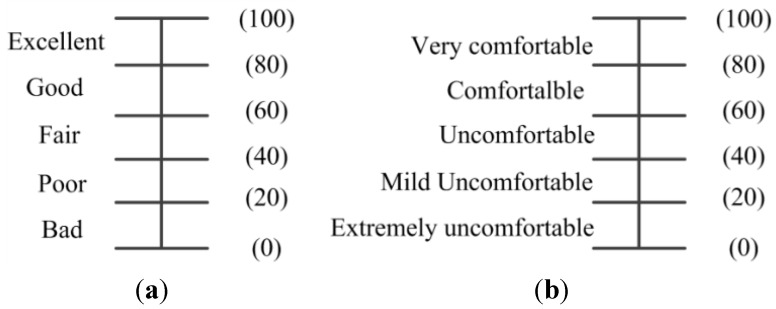
Rating scales used for evaluation. (**a**) Stereoscopic effect; (**b**) temporal smoothness.

Subjective assessment was performed as well. A slightly modified version of single-stimulus presentation method in ITU-R BT.500-11 [19] was used to evaluate the results. The 3D videos synthesized from the aforementioned 2D videos were displayed on the 120 Hz 3D display. Twenty individuals were asked to view the generated stereoscopic videos with active-shutter glasses and rate each video based on two factors: stereoscopic effect and temporal smoothness. The two factors were assessed using a five-segment scale and mapped to a 100 point scale, as is shown in Figure 18.

**Figure 19:**
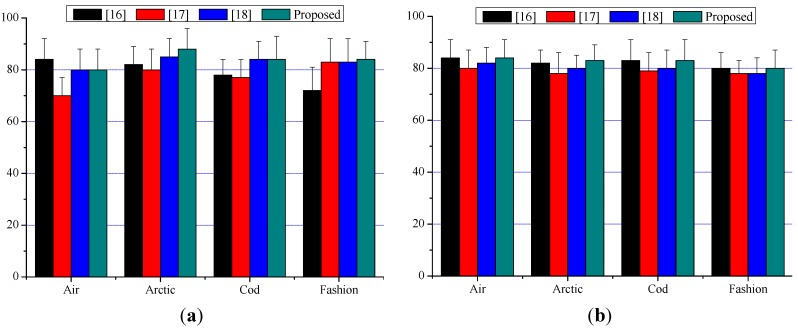
Subjective evaluation results. (**a**) Stereoscopic effect; (**b**) temporal smoothness.

Figure 19 shows the values of the two factors acquired by experiments for the four evaluation video sequences. From the experimental results of subjective assessment, we can find that the proposed algorithm has advantages in stereoscopic effect and temporal smoothness. Better stereoscopic effect is due to the adaptability to different image types of the proposed algorithm. Better temporal smoothness can be attributed to the time-coherent depth maps generated in the process. Thus by using the proposed algorithm, viewers can reduce eye fatigue while they are enjoying stereoscopic videos compared to other algorithms.

Table 3 shows the average processing time of each algorithm for tested video sequences. The spatial resolution of tested videos is 1280 × 720 and the experimental platform is also a PC with Intel quad-core i5 CPU and 4GB RAM. As the reuse of global depth gradient within a video shot reduces computational complexity, the proposed algorithm has an advantage in overall processing time.

**Table 3:** Average Processing time of Different Algorithms.

Algorithm	Average Performance (ms/frame)
[16]	560
[17]	981
[18]	86
Proposed	78

## 4. Conclusions

2D-to-3D video conversion method can make use of 2D video data based on ordinary image sensors to produce 3D video data, which means it can be adopted in a 3D image sensor, which is composed of a general 2D image sensor and the 2D-to-3D chip. This paper presented a novel algorithm for 2D-to-3D video conversion which can be used in the 2D-to-3D chip in the future. The proposed algorithm produces time-coherent depth maps by detecting new video shot frames, generating global depth gradients and supplementing with local depth information. The innovation of generating global depth gradients for different image types can produce good adaptability to different scenes and produce better stereoscopic effects for the generated 3D video. The reuse of global depth gradient can reduce the overall processing time in the 2D-to-3D system. This innovation can also improve the temporal smoothness of generated 3D video, which reduces eye fatigue for viewers and leads to a comfortable visual experience.
